# Mechanisms of Pulmonary Vasculopathy in Acute and Long-Term COVID-19: A Review

**DOI:** 10.3390/ijms25094941

**Published:** 2024-04-30

**Authors:** Marianne Riou, Florence Coste, Alain Meyer, Irina Enache, Samy Talha, Anne Charloux, Cyril Reboul, Bernard Geny

**Affiliations:** 1 Translational Medicine Federation of Strasbourg (FMTS), University of Strasbourg, CRBS, Team 3072 “Mitochondria, Oxidative Stress and Muscle Protection”, 1 rue Eugène Boeckel, CS 60026, 67084 Strasbourg, France; marianne.riou@chru-strasbourg.fr (M.R.); alain.meyer1@chru-strasbourg.fr (A.M.); irina.enache@chru-strasbourg.fr (I.E.); samy.talha@chru-strasbourg.fr (S.T.); anne.charloux@chru-strasbourg.fr (A.C.); 2 Physiology and Functional Exploration Service, University Hospital of Strasbourg, 1 Place de l’hôpital, 67091 Strasbourg, France; 3 EA4278, Laboratoire de Pharm-Ecologie Cardiovasculaire, UFR Sciences Technologies Santé, Pôle Sport et Recherche, 74 rue Louis Pasteur, 84000 Avignon, France; florence.coste@univ-avignon.fr (F.C.); cyril.reboul@univ-avignon.fr (C.R.)

**Keywords:** COVID-19, post-/long COVID, pulmonary vasculopathy, thrombosis, endotheliitis, endothelial dysfunction, pulmonary hypertension

## Abstract

Despite the end of the pandemic, coronavirus disease 2019 (COVID-19) remains a major public health concern. The first waves of the virus led to a better understanding of its pathogenesis, highlighting the fact that there is a specific pulmonary vascular disorder. Indeed, COVID-19 may predispose patients to thrombotic disease in both venous and arterial circulation, and many cases of severe acute pulmonary embolism have been reported. The demonstrated presence of severe acute respiratory syndrome coronavirus 2 (SARS-CoV-2) within the endothelial cells suggests that direct viral effects, in addition to indirect effects of perivascular inflammation and coagulopathy, may contribute to pulmonary vasculopathy in COVID-19. In this review, we discuss the pathological mechanisms leading to pulmonary vascular damage during acute infection, which appear to be mainly related to thromboembolic events, an impaired coagulation cascade, micro- and macrovascular thrombosis, endotheliitis and hypoxic pulmonary vasoconstriction. As many patients develop post-COVID symptoms, including dyspnea, we also discuss the hypothesis of pulmonary vascular damage and pulmonary hypertension as a sequela of the infection, which may be involved in the pathophysiology of long COVID.

## 1. Introduction

The coronavirus disease 2019 (COVID-19) global pandemic, caused by severe acute respiratory syndrome coronavirus 2 (SARS-CoV-2), spread rapidly worldwide in 2020, with more than 750 million people infected and more than 6.9 million deaths over the past three years [1]. In a very short time, we have learned a great deal about this virus, leading to the development of effective vaccines, changes in acute care management and ultimately a reduction in mortality. SARS-CoV-2 angiotensin-converting enzyme 2 (ACE2) receptors are localized on the surface of type I and type II pneumocytes and macrophages, as well as on perivascular pericytes, arterial and venous endothelial cells, and arterial smooth muscle cells. Thus, the lung is the organ most commonly affected by SARS-CoV-2, contributing to the lung damage observed in acute lung injury or acute respiratory distress syndrome (ARDS) and pulmonary vascular damage [2,3,4,5]. Indeed, advances in the understanding of the pathophysiology of COVID-19 have highlighted the key role of the endothelium [6] and, in particular, the pulmonary vascular endothelium [7]. The hyperactivation of the immune system and the release of pro-inflammatory cytokines, the so-called “cytokine storm”, in response to the virus contribute to the lung damage observed in COVID-19 [8].

In addition, one of the major vascular consequences of the disease is coagulopathy, predisposing patients to thrombotic disease in both venous and arterial circulation with pulmonary and extrapulmonary events [9,10,11,12]. As a result, the prognostic value of elevated blood D-dimer levels was recognized early [13], and clinicians were quickly alerted to the risk of thrombosis in these patients. Thus, both intravascular (coagulopathy) and extravascular (endotheliitis and endothelial dysfunction) changes appear to be key mechanisms of the pulmonary vasculopathy observed in COVID-19 patients, and the acronym “AVDS” (for acute vascular distress syndrome) has been proposed to emphasize the vascular target of SARS-CoV-2 [14].

Following acute illness, we have seen the emergence of patients with persistent symptoms/dyspnea, known as long COVID. Given the acute vascular abnormalities identified, there has been concern about long-term pulmonary vascular sequelae and the development of pulmonary hypertension (PH).

We provide a comprehensive review of the acute and long-term pulmonary vascular injury induced via SARS-CoV-2 and its mechanistic considerations.

## 2. Acute Pulmonary Vascular Injury and COVID-19

**Figure 1:**
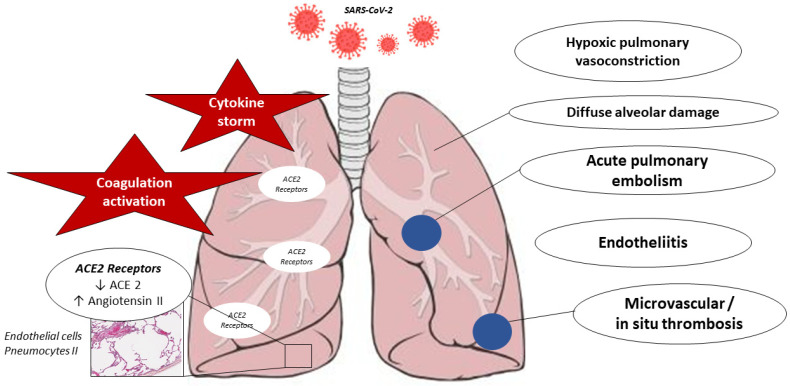
Selected pathophysiological mechanisms involved in COVID-19-associated pulmonary vasculopathy. The SARS-CoV-2 cycle begins with the interaction with the ACE2 (angiotensin-converting enzyme 2) receptor on epithelial cells, macrophages, perivascular pericytes, arterial and venous endothelial cells, and arterial smooth muscle cells.

During acute COVID-19, several mechanisms significantly impair the pulmonary vasculature, including various alterations in the coagulation cascade, such as thromboembolic events, coagulopathy, micro- and macrovascular thrombosis, and endotheliitis and hypoxic pulmonary vasoconstriction (HPV). Figure 1 summarizes the main mechanisms leading to acute pulmonary vascular injury in COVID-19.

### 2.1. COVID-19 and Thromboembolic Events

Since March 2020, early accumulated data have shown an increased risk of thromboembolism in COVID-19, with thrombotic events, particularly pulmonary embolism (PE), being reported as a direct threat to vital prognosis, especially in severe COVID-19, i.e., ARDS [11,15]. These data have since been confirmed by numerous teams around the world [16,17,18,19]. The incidence of PE varies considerably between COVID-19 cohorts, ranging from 0.9 to 3.4% for patients admitted to hospital but not requiring an intensive care unit (ICU) [20,21] and from 8 to 59% for the most severe patients [22,23]. Rates among people not admitted to hospital are lower but still significant [24]. A retrospective, single-center French study showed a 3.4% incidence of PE in 1696 patients who underwent a chest CT scan for suspected or follow-up COVID-19. Of these patients, 106 were injected for suspected PE, with PE identified in 30% of cases [9]. These observations have been strengthened by the fact that one of the first studies to describe the results of complete autopsies performed on 12 patients who died from COVID-19 in Germany revealed unexpectedly severe thrombotic damage [25]. Massive PE with deep vein thrombosis (DVT) was found to be the cause of death in 33% (4 out of 12) of cases, although this diagnosis had not been suspected before death. In three other cases, the patients had DVT but no PE. Additionally, in all these patients, the DVT was bilateral. Subsequently, many anatomopathological studies have found similar results [26]. Interestingly, the risk of PE continues into the non-acute phase: the pooled cumulative incidence across studies is 1–5% during short-term follow-up [27], and it remains elevated for 8 weeks or more after diagnosis [28].

Understanding the coagulopathy induced via SARS-CoV-2 has, therefore, become crucial to diagnosing it as early as possible and optimizing thromboprophylaxis management. As the rate of venous thromboembolic events (VTE) was significantly higher in patients requiring intensive care [29], a distinction was made between these patients (whose thromboembolic risk was clearly increased) and less severely affected patients. In a retrospective cohort of 184 patients admitted to ICU in the Netherlands for COVID-19, the cumulative probability of VTE was 31% despite thromboprophylaxis at approximately 2 weeks’ follow-up in the absence of a systematic screening strategy [30]. This high prevalence could be explained using several hypotheses: During the first phase of the pandemic, COVID-associated endothelial damage and coagulopathy, potentialized via the often-prolonged immobilization of critically ill patients, were recognized as key players in the prothrombotic profile of COVID-19. In addition, significant risk factors associated with VTE in COVID-19 have been identified, including the male sex, an older age, the receipt of mechanical ventilation, and elevated C-reactive protein and D-dimer [31,32]. Clinically, this has led not only to conventional VTE (PE, DVT) but also to arterial macrothrombosis, including ischemic stroke, and local (immune) microthrombosis, particularly in the pulmonary vasculature. Thrombosis of extracorporeal purifying filters and extracorporeal membrane oxygenation (ECMO) have also been observed. These data were even more worrying, as the majority of ICU patients were receiving anticoagulant treatment with at least standard low-dose anticoagulation [30]. Several studies have subsequently investigated the potential excess risk of thromboembolic events in COVID patients compared to other viral infections. Ultimately, the risk of VTE in COVID patients was found to be similar to that in other patients, with the exception of the most severely ill patients hospitalized in an ICU [33]. In any case, the prevalence of VTE was the highest during the first wave, with a much smaller increase during the second wave, highlighting the results of more appropriate care, i.e., adaptive anticoagulation in the most severe COVID-19 patients [34,35].

### 2.2. COVID-19 and Coagulopathy

Routinely measured hemostasis parameters such as the platelet count, activated partial thromboplastin time (APTT), prothrombin rate and international normalized ratio (INR) remained within normal limits in the majority of COVID-19 patients, including the most severe patients, whether in the ICU or with thrombotic events [12,16,36]. More interestingly, elevated D-dimer levels have been described and correlated with disease severity [13,37]. For example, in a large clinical case analysis of 1099 patients with confirmed COVID-19 from more than 550 hospitals in China, 46.4% of patients had D-dimer levels > 0.5 mg/L, including 60% with severe disease [36]. This was also true for non-severe COVID-19 patients (43%). This was confirmed by Huang and colleagues, who showed that D-dimer levels on admission were higher in ICU patients (median D-dimer level: 2.4 mg/L) than in other patients (median D-dimer level: 0.5 mg/L) [38]. Taken together, these data suggest that elevated D-dimer levels indirectly reflect the presence of coagulopathy and are associated with increased morbidity and mortality in hospitalized COVID-19 patients. D-dimers are the specific degradation products of fibrin polymers stabilized via Factor XIIIa under the action of plasmin and are, thus, a marker of fibrin formation and degradation. An elevated level may, therefore, reflect the pathological activation of coagulation in COVID-19 patients. Rather than an isolated value, the kinetics of the D-dimer level appear to be more relevant [13,38,39,40,41].

As in sepsis, fibrinogen (coagulation factor I) levels are greatly increased in COVID-19 patients, especially in the most severe forms. Its elevation also correlates with thrombotic risk. In addition, many authors have suggested a role for lupus anticoagulant (LA) associated with factor XII deficiency in the thrombotic manifestations associated with severe COVID-19 [16,42,43,44]. The presence of LA may be associated with a thrombotic tendency within the antiphospholipid syndrome, but it has also been described in many other pathologies, particularly infectious pathologies, and even in the elderly, and its generation follows cellular damage or activation secondary to an infectious, autoimmune, inflammatory or drug trigger [45]. However, the pathogenic role of LA in COVID-19 has not yet been established [46].

Numerous studies have also found extremely high levels of factor VIII and von Willebrand factor (vWF), indicating endothelial activation [16]. vWF is synthesized via endothelial cells or megakaryocytes and is either secreted into the plasma or stored in intercellular organelles (Weibel–Palade bodies). It is involved in the modulation of platelet adhesion and aggregation. One hypothesis to explain the increased vWF levels in COVID-19 is the release of vWF from Weibel–Palade storage bodies after the virus’s entry into endothelial cells, mediating platelet aggregation and adhesion. In addition, reduced levels of the specific vWF-cleaving protease ADAMTS 13 (a disintegrin and metalloprotease with a thrombospondin type 1 motif member 13) have been described in COVID-19. Plasma levels of soluble P-selectin, derived from activated platelets or following secretion from Weibel–Palade bodies, are also significantly elevated in patients with severe COVID-19 [47].

In addition, some evidence has shown an association between coagulopathy and liver damage in COVID-19 [48]. Liver dysfunction with elevated transaminase levels is commonly observed in patients with COVID-19. SARS-CoV-2 may induce liver injury due to the high expression of ACE2 in cholangiocytes, which may be potentiated by certain drugs used to treat COVID-19 such as antibiotics, remdesivir, tocilizumab, tofacitinib or dexamethasone [49,50]. Interestingly, liver microvascular thrombosis has been described in postmortem findings [51]. However, it remains unclear whether liver injury may contribute to the abnormalities in coagulation and hemostasis observed in COVID-19.

Finally, several teams have found abnormalities in viscoelastic tests such as thromboelastography (TEG^®^), thromboelastometry (ROTEM^®^) or sonoheometry (Quantra^®^) in severe COVID-19 patients. These tests, which can be performed “at the patient’s bedside”, allow an analysis of the viscoelastic properties of the clot throughout the coagulation process and, unlike conventional coagulation tests, can detect hyperfibrinolysis [37,41,52]. In this context, it may be useful to better explore hypercoagulability and predict thrombotic events.

However, the coagulopathy disorders induced via COVID-19 are not those expected in sepsis [37]. While 30–40% of septic shock patients develop disseminated intravascular coagulation (DIC) [53], the majority of COVID-19 cases did not [54,55,56].

### 2.3. Mechanisms Implicated in Micro- and Macrovascular Thrombosis

Several processes through which SARS-CoV-2 infection may lead to microvascular and macrovascular thrombi have been suggested, including the hyperinflammation/dysregulation of immune cells, endothelial and platelet function, hypoxia and direct viral effects with cell activation.

Severe COVID-19 is associated with a cytokine storm characterized by elevated plasma concentrations of interleukins (IL-1ß, IL-2, IL-6, IL-7, IL-8, IL-10 and IL-17), interferons, monocyte chemoattractant protein 1 (MCP-1) and TNF-alpha [57]. Cytokines can activate vascular endothelial cells and cause endothelial injury, leading to prothrombotic properties [58]. Circulating levels of IL-6 have been described as an important indicator of mortality in severe COVID-19 [59]. In addition, reactive oxygen species (ROS) induced via hypoxemia and/or viral infection are key signaling molecules that play an important role in the progression of inflammatory disorders, resulting in oxidative stress, mitochondrial dysfunction and DNA damage, and they may also be involved in the pulmonary vascular disorders observed in COVID-19 [60,61]. Autopsy reports have also suggested a possible role for complement and neutrophil activation in the thrombus formation observed in COVID-19 [62,63]. Indeed, microvascular thrombi contained numerous neutrophils, some of which were partially degenerated, consistent with neutrophil extracellular traps (NETs) [64]. NETs are web-like structures of DNA and proteins shed by neutrophils that interact with pathogens. Although NETs are beneficial in the host’s defense against pathogens, excessive NET formation can stimulate an inflammatory response or intravascular thrombosis [65,66]. Platelet–leukocyte complexes have also been isolated from the circulation of COVID-19 patients. These complexes show the upregulation of tissue factor (TF) expression, which further contributes to the hypercoagulable state observed in COVID-19 patients [67].

COVID-19-induced endothelial cell dysfunction is another mechanism involved in thrombosis [68]. Once infected with SARS-CoV-2 via ACE2, the activated or damaged endothelial cells release vWF from their Weibel–Palade granules into the bloodstream (FVIII is not contained in the granules but is secondarily increased due to its plasma transport via vWF). vWF acts as a “bridge” (1) between activated platelets and damaged endothelial cells or the subendothelium and (2) between the platelets themselves. The infection and inflammation of the endothelium (called endotheliitis) lead to endothelial dysfunction and is also involved in the expression of TF [56]. In addition, the cytokine storm (described above) due to hyperinflammation induces a pro-adhesive state, mainly by increasing the expression of adhesion proteins such as ICAM1 and VCAM1, and a procoagulant state of the endothelium, thus favoring thrombosis [69].

Pulmonary inflammation induced via SARS-CoV-2 impairs fibrinolytic function and leads to an abnormal alveolar accumulation of fibrin, as described in broncho-alveolar lavage from COVID-19 patients. In the healthy lung, there is a balance between host coagulation and fibrinolysis that allows for the fine control of fibrin deposition and its influence on pulmonary epithelial viability; urokinase plasminogen activator (uPA) linked to its receptor (uPAR) increases the efficiency of fibrinolysis on the surface of epithelial cells, thereby eliminating abnormal fibrin deposits in the lung [70]. Menter et al. reported a postmortem evaluation of 10 COVID-19 patients and the presence of diffuse alveolar damage and fibrin-platelet thrombi in small pulmonary arteries [71].

As ACE2 is a major component of the renin–angiotensin–aldosterone system (RAAS), this system is also implicated in the pathophysiology and coagulopathy of COVID-19 [72]. Angiotensin II induces tissue factor and plasminogen activator inhibitor-1 (PAI-1) expression in endothelial cells via the angiotensin-1 receptor, contributing to a hypercoagulable state [73].

Finally, hypoxemia could also induce a hypercoagulable state not only by increasing blood viscosity but also through a hypoxia-inducible transcription factor-dependent signaling pathway [74].

### 2.4. COVID-19 and Endotheliitis

Under normal conditions, the vascular endothelium plays a vital physiological role, controlling vascular tone, inflammation, oxidative stress and vascular permeability and providing an antithrombotic surface by expressing endogenous molecules that inhibit both platelet activation (e.g., nitric oxide (NO), prostacyclin and ectonucleotidases) and coagulation (e.g., tissue factor pathway inhibitor (TFPI), thrombomodulin and endothelial cell protein C receptor (EPCR)) [75]. Endothelial cells express the Tie2 receptor tyrosine kinase, which blocks the action of inflammatory cytokines by inhibiting nuclear factor kappa beta (NF-kB) signaling from activated B cells [76]. The endothelium also secretes a glycocalyx—composed of proteoglycans and glycosaminoglycans—which reduces water permeability, inhibits the entry of plasma proteins and has an antithrombotic function. In the lung, the vascular endothelium is the critical barrier between pulmonary circulation and the interstitium.

Endothelial cell injury or activation is a central feature of the pathogenesis of COVID-19, potentially due to many mechanisms: the direct cytotoxicity of SARS-CoV-2 or indirect effects via a cytokine storm or other innate, adaptive or autoimmune phenomena [7,77]. Electron microscopy studies have reported SARS-CoV-2 virus particles within the endothelium of various organs, particularly during the inflammatory phase of the disease [77]. Although ACE2 receptors are expressed at lower levels on the endothelial surface than on epithelial cells [78], the ACE2 receptor and the serine protease TMPRSS2, expressed via the endothelial membrane, are likely to be the entry points for the virus [4]. In addition, other endothelial cell receptors mediate SARS-CoV-2’s entry, such as neuropilin-1, CD147 and CD26 [79,80]. Endothelial cell dysfunction in COVID-19 may result in increased vWF, platelet activation and hypercoagulability, as described above [47]. Furthermore, an impaired microcirculatory function was objectified by measuring altered sublingual microvascular perfusion [81].

In addition, as mentioned above, the inflammatory response induced via SARS-CoV-2 is orchestrated through the endothelium. In response to viral pathogen-associated molecular patterns (PAMPs) and immune cell activation, the endothelial cells secrete inflammatory cytokines such as IL-1β, IL-18, IL-6, IL-8 and TNF-α. As a result, the endothelium loses its antithrombotic and anticoagulant functions, loses its glycocalyx (due to the secretion of proteases) and increases endothelial ROS production [82]. Endothelial oxidative stress, with consequent reduced endogenous NO bioavailability, has emerged as a likely pathogenic factor in endothelial dysfunction among ICU COVID-19 patients [83]. Under physiological conditions, NO is produced in small amounts via the endothelial isoform of the nitric oxide synthase (eNOS), which induces the synthesis of cyclic guanosine monophosphate (cGMP) with consequent vasodilatory, antioxidant, antimicrobial and antithrombotic effects. The NO pathway plays a key role in the regulation of vascular function throughout the vascular tree, particularly in the pulmonary vasculature. Endothelial dysfunction and increased thrombogenicity classically result from an imbalance between NO produced via the eNOS and ROS produced through NADPH oxidase (NOX)-2 or uncoupled eNOS. Montiel et al. showed that COVID-19 patients had reduced 5-α-nitrosyl-hemoglobin (HbNO), reflecting decreased vascular NO bioavailability, which was proportional to their disease severity [83]. Such an alteration may contribute to the microvascular dysfunction observed in COVID-19 patients and the thrombotic microangiopathy that complicates ICU COVID-19. Conversely, in this study, patients suffering from septic shock with a cytokine-dependent induction of the inducible NO synthase (iNOS) showed increased HbNO levels.

In contrast to the first SARS epidemic and what is typically seen in diffuse alveolar damage, distinctive pulmonary vascular pathological features have been described in COVID-19; see Table 1 [5,7]. Autopsies of patients with COVID-19 have highlighted the role of inflammation, endothelial cell damage and platelet dysfunction in SARS-CoV-2-induced pulmonary vascular coagulopathy (Table 1). A histological examination of the lungs in autopsy series not only confirmed diffuse alveolar damage (necrosis of alveolar lining cells, pneumocyte-2 hyperplasia and linear intra-alveolar fibrin deposition) but also showed widespread microthrombi present within peripheral small vessels (arteries and venules) and capillaries [62,84]. Inflammatory cell infiltrates consisting of CD4+ and CD8 + lymphocytes have been described in the interstitial spaces and around larger bronchioles and blood vessels [62]. In addition, aggregates of CD4+ were observed around the small vessels, some of which appeared to contain platelets and small thrombi. Alveolar capillaries were markedly thickened with surrounding edema, and fibrin thrombi were present within the capillaries and small vessels and often associated with foci of alveolar hemorrhage. Cell growth via the activation of the mitogen-activated protein kinase (MEK)/extracellular signal-regulated kinase (ERK) pathway via the SARS-CoV-2 spike protein may be one of the mechanisms leading to capillary thickening [5]. Many CD61+ megakaryocytes, possibly lung-native, were present within the small vessels and alveolar capillaries and may contribute to inflammatory recruitment and platelet activation, leading to thrombus formation. In addition, severe endothelial injury associated with intracellular SARS-CoV-2 virus and disrupted endothelial cell membranes has been described in the lungs of dead patients with COVID-19, suggesting a direct viral effect on endothelial dysfunction [7]. Finally, a mechanism of intussusceptive (non-sprouting) angiogenesis leading to new vessel growth has been observed in the lungs of deceased patients with COVID-19 [7]. Indeed, distorted vascularity with structurally deformed and elongated capillaries with sudden changes in caliber and the presence of intussusceptive pillars within the capillaries has been observed in the lungs of patients with COVID-19. Intussusceptive angiogenesis is a dynamic intravascular process that induces an important change in the structure of the microcirculation that cannot be seen using conventional light microscopy, but the mechanisms are unclear [85]. In most cases, angiogenesis is due to a mechanism called “sprouting”, which results in the proliferation of endothelial cells from the vessel’s endpoints and walls, connecting vessels to each other. However, this mechanism was not predominant in COVID-19. Intussusception was mainly observed due to endothelial cell proliferation within the vessels. Interestingly, the degree of intussusceptive angiogenesis in COVID-19 correlated with the duration of hospitalization [7].

**Figure 2:**
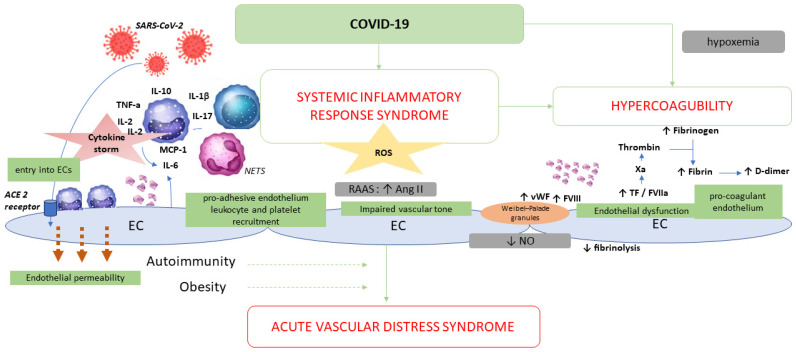
Schematic representation of the mechanisms through which SARS-CoV-2 induces hypercoagulability and endotheliitis.

Mechanisms involved in pulmonary vascular thrombosis and endothelial dysfunction are summarized in Figure 2.

COVID-19 induces uncontrolled inflammation, a so-called cytokine storm, including IL-6, IL-1b, IL-2, IL-10, TNF-α and monocyte chemoattractant protein (MCP-1). It induces the following: (1) endothelial dysfunction, (2) an impaired endothelial regulation of vascular tone and (3) microthrombosis and the occlusion of small pulmonary vessels.

ACE2: angiotensin-converting enzyme 2; Ang: angiotensin; EC(s): endothelial cell(s); IL: interleukin; NETs: neutrophil extracellular traps; NO: nitric oxide; RAAS: renin–angiotensin–aldosterone system; ROS: reactive oxygen species; TF: tissue factor; and TNF: tumor necrosis factor.

### 2.5. Hypoxic Pulmonary Vasoconstriction (HPV)

Apart from COVID-19, it has been well demonstrated that deep hypoxemia in patients plays a role in the pulmonary capillaries, causing HPV, which reduces blood flow and promotes vascular occlusion [103]. In healthy lungs, HPV is the homeostatic response of the pulmonary circulation to ensure an optimal gas exchange, resulting in a redistribution of perfusion (Q) to the better-ventilated area (V), thus optimizing V/Q matching (V/Q = 1). As described by Archer et al., COVID-19 hypoxemia is multifactorial and can be attributed to ARDS, impaired HPV and a high altitude pulmonary edema physiology [104]. In ARDS, blood flow redistribution is severely impaired, characterized by areas of high perfusion and low ventilation (V/Q < 1, where V/Q = 0 is termed intrapulmonary shunting) and areas of high ventilation and low perfusion (V/Q > 1, where V/Q∼∞ is termed dead space ventilation) [105]. In acute COVID-19, Gattinoni et al. have suggested that shunt values may be so elevated as to be considered “excessive” for the degree of lung injury present [106]. This suggests that SARS-CoV-2 may specifically affect HPV (in addition to intravascular occlusion via microthrombi, the extravascular compression of vessels due to edema and atelectasis, and interstitial fibrosis) compared to other causes of ARDS.

Hypoxia also induces the activation of hypoxia-inducible factors (HIFs) [107]. HIFs are heterodimeric transcription factors consisting of the HIF-β subunit, which is expressed in all nucleated cells, and the HIF-α 1 and 2 subunits. Under normoxic conditions, HIF-α is hydroxylated and degraded. In contrast, under hypoxic conditions, HIF-α is not degraded, and it translocates to the nucleus, where it binds to HIF-β and initiates gene transcription. This results in the upregulation of a large number of target genes that contribute to hypoxia adaptation, including the erythropoietin (EPO) gene (leading to the production of more red blood cells) and VEGF (leading to the production of more blood vessels). Interestingly, VEGF-A would be a relevant predictor of diffusing the lung capacity for carbon monoxide (DLCO) impairment and radiological sequelae at a distance from infection [108].

## 3. Long COVID-19-Related Pulmonary Vasculopathy

Many individuals have reported symptoms persisting for three months or more, lasting at least two months after acute SARS-CoV-2 infection, referred to as the long COVID or post-COVID-19 condition, recognizing that these symptoms are not attributed to other diseases [109]. This is an important public health issue, as demonstrated by Van Wambeke et al., who found that one third of patients had persistent symptoms and had not returned to work as late as 22 months after their COVID infections [110]. This clinical syndrome is heterogeneous and can affect multiple organ systems, with breathlessness being one of the most common symptoms [111]. Parenchymal pulmonary sequelae, i.e., pulmonary fibrosis, muscle-related breathlessness and/or extrapulmonary sequelae, are likely to contribute to dyspnea [112,113,114] but are beyond the scope of this review.

Furthermore, given the factors described above, it is likely that acute pulmonary vasculopathy (i.e., VTE, small in situ pulmonary thrombi, the deterioration of capillary integrity and endotheliitis) is one of the mechanisms potentially responsible for longer-term sequelae.

In addition, several pathophysiological aspects link COVID-19 and pulmonary arterial hypertension (PAH), such as the role of ACE2 or pulmonary endothelial dysfunction [115]. Moreover, systemic RAAS activity is increased in idiopathic PAH patients and is associated with disease progression and mortality, and angiotensin II induces pulmonary artery smooth muscle cell proliferation via angiotensin-1 receptor signaling [116]. PAH is a rare, progressive vascular disease that leads to the vascular remodeling of the small pulmonary arteries, right heart failure and ultimately death. It results in elevated pulmonary arterial pressure (PAP) and pulmonary vascular resistance (PVR) as measured via right heart catheterization (RHC) with mean PAP > 20 mmHg, pulmonary capillary wedge pressure ≤ 15 mmHg and PVR > 2 Wood units [117].

**Figure 3:**
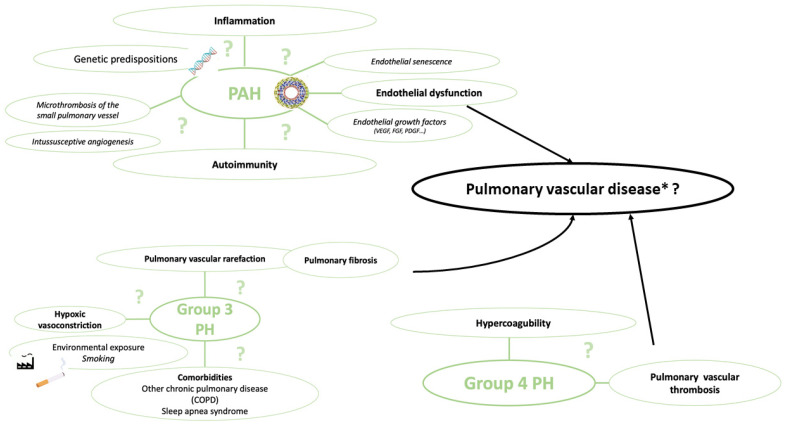
Possible mechanisms of pulmonary vascular disease after COVID-19. * Pulmonary vascular disease is defined as precapillary pulmonary hypertension (PH), in turn defined by right heart catheterization as mean pulmonary artery pressure > 20 mmHg, pulmonary capillary wedge pressure ≤ 15 mmHg and pulmonary vascular resistance > 2 Wood units. COPD: chronic obstructive pulmonary disease, FGF: fibroblast growth factor; group 3 PH: pulmonary disease associated with respiratory disease; group 4 PH: chronic thromboembolic pulmonary hypertension; PDGF: platelet-derived growth factor; PH: pulmonary hypertension; and VEGF: vascular endothelial growth factor.

Additionally, due to acute pulmonary parenchymal lesions and the incidence of PE, COVID-19 survivors are also exposed to group 3 PH due to parenchymal sequelae (pulmonary fibrosis) or chronic thromboembolic pulmonary hypertension (CTEPH); see Figure 3.

Moreover, other viruses have been implicated in the development of pulmonary vascular disease over the years, such as human immunodeficiency virus (HIV), which has been associated with PAH [118]. Human herpesvirus-8 (HHV-8) infection has been described in the plexiform lesions (e.g., local production of inflammatory cytokines) and lung parenchymal cells of patients with PAH, but the role of HHV8 in PAH pathophysiology is controversial [119,120].

### 3.1. Chronic Thromboembolic Pulmonary Hypertension (CTEPH)

At the onset of the pandemic, there was a growing awareness of an increased risk of chronic thromboembolic disease in COVID-19 survivors. Indeed, given the pooled incidence rate of PE of 16.5% reported in COVID-19 [121] and the mechanisms of pulmonary coagulopathy, it could be hypothesized that there would be less thrombus resolution after PE associated with COVID-19 and that the prevalence of CTEPH would be higher compared to populations with PE not associated with COVID-19. Furthermore, multiomics analysis revealed the persistent upregulation of gene expression signatures related to vascular and coagulation pathways several months after infection, elucidating the biological basis for the long-lasting prothrombotic state associated with COVID-19 [122]. Some studies have investigated the rate of thrombosis after discharge from hospital. Venturelli et al. reported dyspnea in 51.4% of 767 COVID-19 patients after discharge, with 17% having elevated D-dimer levels, including 2 who were found to have silent pulmonary thrombosis [123]. In another retrospective study of 163 patients, the cumulative incidence of arterial or venous thrombosis at 30 days after discharge was 2.5% and 0.6% for VTE alone [124]. More recently, Caguana-Vélez et al. reported an incidence of chronic thromboembolic pulmonary disease of 0.75% after 2 years of follow-up in 133 COVID-19 patients [125]. In any case, if PE is diagnosed during acute illness or at follow-up, patients should be treated according to PE guidelines [126].

In a non-pandemic setting, CTEPH occurs in approximately 1–2% of patients after acute PE. However, an increase in the incidence of CTEPH after COVID-19 has not been reported. Newman et al. reported the results at a national specialist center in the United Kingdom and found no increase in the incidence of CTEPH over 12 months; nor did they detect a single case that was clearly associated with COVID-19 [127]. Recently, de Jong and colleagues confirmed this hypothesis, as no CTEPH was diagnosed, and 4.4% of 209 patients had residual thrombotic obstruction at 6 months [128].

Some studies have reported transthoracic echocardiographic right ventricular dysfunction and suspected PH in COVID-19 patients, but it seems incomplete to draw a conclusion, especially as no RHC was performed [129]. Rossi et al. demonstrated mild PH during the acute phase of COVID-19 (in hospitalized patients), confirmed via RHC, with progressive recovery of RV function and PH after 6 months and 1 year of follow-up [130]. In any case, in COVID-19 patients with persistent exertional dyspnea without evidence of parenchymal lung opacities on high-resolution CT 3–6 months after discharge and with pulmonary function tests documenting preserved lung volumes and normal or reduced DLCO, follow-up evaluation should include echocardiography and contrast-enhanced CT to detect significant pulmonary vascular involvement, according to proposed guidelines [131,132]. However, contrast-enhanced CT is not always sufficient to exclude CTEPH [133], and lung perfusion imaging with ventilation–perfusion (VQ) planar scintigraphy, VQ single-photon emission computed tomography (SPECT) or contrast dual-energy CT (DECT) is warranted to assess residual blood flow limitations in symptomatic COVID-19 patients, even in the absence of a history of PE during acute illness [134]. In this case, the patient should be referred to a specialized PH center, as recommended [117].

### 3.2. Persistent Vascular Endothelial Dysfunction

Persistent vascular endothelial dysfunction appears to be common in patients who remain symptomatic after the acute phase of COVID-19, suggesting that it may be involved in the pathogenesis of long COVID syndrome [135]. Indeed, although not always directly related to SARS-CoV-2, systemic endothelial dysfunction has been observed in several vascular beds more than 3 months after the onset of infection [136,137,138]. Regarding the pulmonary vascular bed, Zhou et al. demonstrated reduced pulmonary microvascular perfusion in 10 COVID-19 patients (8 months after infection) compared to age-matched healthy volunteers using dynamic performed contrast-enhanced magnetic resonance imaging (DCE-MRI) [139]. Interestingly, as in PAH [140], increased plasmatic levels of the prothrombotic protein NEDD9 have been described in these patients, reflecting pulmonary endothelial dysfunction [141].

In addition, oxidative stress and mitochondrial dysfunction are involved in the endothelial dysfunction observed in acute COVID-19 [142,143] and may contribute to long COVID [144]. Interestingly, as mitochondrial dysfunction is involved in pulmonary vascular remodeling and endothelial dysfunction in PAH, it may be another potential mechanism of long-term pulmonary sequelae after COVID-19 [145,146].

Furthermore, as thrombi or microthrombi are more commonly reported in autopsy studies, they may be involved in the post-acute morbidity associated with COVID-19 [87]. Halawa et al. highlighted the similarities between COVID-19-induced pulmonary vascular injury and PAH/CTEPH, including endothelial cells, vascular smooth muscle cells, fibroblasts and pericytes [147]. Moreover, osteopontin (OPN), a matricellular protein, is involved in the inflammatory response to infection and in thrombosis driven by chronic inflammation, making it a potential biomarker for acute and long COVID-19 [148,149]. In the longer term, it may contribute to pulmonary vascular remodeling, as elevated circulating levels of OPN have been associated with severity in idiopathic PAH [150,151]. In addition, a worse outcome has been described in PAH patients infected with SARS-CoV-2 (23% in-hospital mortality rate), suggesting that COVID-19 may act as a second hit for the worsening of pulmonary endothelial dysfunction due to all the mechanisms described above [152]. However, despite the pathobiological link between PAH and COVID-19, there is currently no evidence of an increase in PAH incidence following COVID-19. Prospective studies considering COVID-19 in the medical history of a new case of PAH will be interesting to better understand the potential role of infection as a trigger for the development of PAH.

Finally, the different SARS-CoV-2 variants also contribute to variable viral transmission, disease severity and vaccine efficacy, potentially leading to heterogeneity in pulmonary vascular (endothelial) damage. Hamsters inoculated with SARS-CoV-2 Omicron had milder pulmonary endothelial effects compared to other variants [153]. In humans, among SARS-CoV-2 variants, patients infected with Delta had the highest risk of ICU admission and mortality, suggesting that this variant is more likely to induce endothelial dysfunction [154]. In addition, effective vaccination against SARS-CoV-2 has reduced the severity of acute infection.

## 4. Conclusions

The first waves of COVID improved our understanding of the pathogenesis of the virus and highlighted the associated pulmonary vascular damage. With the constant mutation of the virus and the new variants, vaccination and changes in the management of acute infection, acute vasculopathy associated with the virus appears to be less common. As highlighted in this review, the pulmonary vascular damage described in COVID-19 is likely to be multifactorial, involving some degree of VTE, impaired coagulation cascade, micro- and macrovascular thrombosis, endotheliitis and endothelial dysfunction, and modified HPV. COVID-19 may, therefore, predispose non-PAH patients to the development of PAH or CTEPH development, but to date, there is no solid evidence to support this, particularly in long COVID patients. When in doubt, patients should be referred to an expert PH center for echocardiography, further imaging and exercise testing to better understand the mechanisms involved, and RHC should be discussed if PH is suspected [117,155]. Nevertheless, further prospective studies focusing on the long-term outcome of these patients are needed, particularly regarding the risk of developing PH in the future.

## Figures and Tables

**Table 1:** Pulmonary vascular findings in the main autopsy series of patients with COVID-19.

Authors	Number of Patients	Lung Histology
[86]	N = 1	- Diffuse alveolar damage - Abundant thrombi in medium and small-sized blood vessels
Ackermann, N Engl J Med. 2020 [7]	N = 7	- Diffuse alveolar damage - Perivascular T-cell infiltration - Severe endothelial injury: intracellular virus and disrupted cell membranes - Pulmonary vessels thrombosis - Microangiopathy (alveolar capillary microthrombi) - Intussusceptive angiogenesis
Borczuk, Mod Pathol. 2020 [87]	N = 68	- Diffuse alveolar damage - Large vessel thrombi (42%) - Platelet and/or fibrin microthrombi (84%) - Small vessels: basal membrane reduplication and significant endothelial swelling with cytoplasmic vacuolization
Bösmüller, Virchows Arch, 2020 [88]	N = 4	- Endotheliitis: multiple microthrombi - Diffuse alveolar damage
Bradley, Lancet. 2020 [89]	N = 14	- Diffuse alveolar damage (acute or organizing phase) - Focal pulmonary microthrombi (N = 5)
De Michele, Am J Clin Pathol, 2020. [90]	N = 40	- Acute lung injury (73%) - Intravascular fibrin or platelet-rich aggregates (90%) - Hemangiomatosis-like change (50%)
Elsoukkary, Patholiology, 2020 [91]	N = 32	- Large vessel thrombi (34%), small vessel thrombi (72%) or both (28%) - Exsudative and proliferative diffuse alveolar damage - Organizing pneumonia
Fox, Lancet Respir Med 2020 [62]	N = 10	- Thrombosis and microangiopathy in the small vessels and capillaries - Diffuse alveolar damage: hyaline membranes
Hanley, Lancet Microbe. 2020 [92]	N = 9	- Thrombosis (89%) - Diffuse alveolar damage
Kianzad, Respirology 2021 [93]	N = 8	- Diffuse alveolar damage +/− microvascular damage and thrombosis
Lax, Ann Intern Med. 2020 [94]	N = 10	- Diffuse alveolar damage - Thrombosis of small and mid-sized pulmonary arteries (+infarction in eight patients)
Luo, 2020 [95]	N = 1	- Extensive pulmonary interstitial fibrosis - Alveolitis - Necrotizing bronchiolitis - Pulmonary hemorrhagic infarct - Hyperplasia, wall thickening and lumen stenosis/occlusion in small vessels - Interstitial infiltration of inflammatory cells
Menter, Histopathology. 2020 [71]	N = 21	- Exudative diffuse alveolar damage - Massive capillary congestion with microthrombi - Pulmonary embolism (N = 4) - Alveolar Hemorrhage (N = 3) - Vasculitis (N = 1)
Miggiolaro, Viruses 2023 [96]	N = 24	- Activated endothelial cells and high levels of ICAM-1, Angiopoietin-2, IL-1β) - Microthrombosis - Tissue expression of VEGF and VEGFR-1
Rapkiewicz, EClinicalMedicine. 2020 [97]	N = 7	- Pulmonary arteries thrombi (N = 4) - Megakaryocytes and platelet-rich thrombi in the lungs
Schaller, JAMA. 2020 [98]	N = 10	- Acute and organizing diffuse alveolar damage
Tian, J Thorac Oncol, 2020 [99]	N = 2	- Edema - Proteinaceous exudate - Inflammation: fibrinoid material and multinucleated giant cells
Varga, Lancet. 2020 [77]	N = 3	- Viral elements within endothelial cells
Wichmann, Ann Intern Med. 2020 [25]	N = 12	- Massive pulmonary embolism (N = 4) - Diffuse alveolar damage: hyaline membranes and activated pneumocytes - Microvascular thromboemboli - Capillary congestion - Protein-enriched interstitial edema - Inflammatory infiltrate (lymphocytes) - Squamous metaplasia in the end stages
Xu, Lancet Respir Med. 2020 [8]	N = 1	- Bilateral diffuse alveolar damage - Hyaline membrane formation - Interstitial lymphocytar infiltrates - Enlarged pneumocytes
Yao, Cell Res 2020 [100]	N = 1	- Diffuse alveolar damage - Formation of hyaline membranes in the alveolar space - Thickening of the alveolar septa - Interstitial inflammation - Thrombus in the microvessels - No pulmonary edema
Youd, J Clin Pathol. 2020 [101]	N = 9	- Diffuse alveolar damage
Zhang, Ann Intern Med, 2020 [102]	N = 1	- Diffuse alveolar damage - Type II pneumocyte hyperplasia - Chronic inflammatory infiltrates - Intra-alveolar fibrinous exudates + organizing fibrin

## Abbreviations

ACE2	angiotensin-converting enzyme 2
APTT	activated partial thromboplastin time
ARDS	acute respiratory distress syndrome
AVDS	acute vascular distress syndrome
cGMP	cyclic guanosine monophosphate
COVID-19	coronavirus disease 2019
CT	computed tomography
CTEPH	chronic thromboembolic pulmonary hypertension
DECT	contrast dual-energy CT
DLCO	diffusing capacity for carbon monoxide
DVT	deep vein thrombosis
ECMO	extracorporeal membrane oxygenation
eNOS	endothelial nitric oxide synthase
EPCR	thrombomodulin and endothelial cell protein C receptor
EPO	erythropoietin
ERK	extracellular signal-regulated kinase
HbNO	5-α-nitrosyl-hemoglobin
HHV-8	human herpesvirus-8
HIF	hypoxia-inducible factor
HIV	human immunodeficiency virus
HPV	hypoxic pulmonary vasoconstriction
ICU	intensive care unit
IL	interleukin
INR	international normalized ratio
iNOS	inducible NO synthase
LA	lupus anticoagulant
MCP-1	monocyte chemoattractant protein 1
MEK	mitogen-activated protein kinase
NETs	neutrophil extracellular traps
NF-kB	nuclear factor kappa beta
NO	nitric oxide
OPN	osteopontin
PAI-1	plasminogen activator inhibitor-1
PAH	pulmonary arterial hypertension
PAMPs	pathogen-associated molecular patterns
PAP	pulmonary arterial pressure
PE	pulmonary embolism
PH	pulmonary hypertension
PVR	pulmonary vascular resistance
RAAS	renin–angiotensin–aldosterone system
RHC	right heart catheterization
ROS	reactive oxygen species
SARS-CoV-2	severe acute respiratory syndrome coronavirus 2
SPECT	ventilation-perfusion single-photon emission computed tomography (SPECT)
TF	tissue factor
TFPI	tissue factor pathway inhibitor
uPA	urokinase plasminogen activator
uPAR	urokinase plasminogen activator receptor
VEGF	vascular endothelial growth factor
VTE	venous thromboembolic events
vWF	von Willebrand factor
